# Supplementary material for: A Method for the Analysis of Glyphosate, Aminomethylphosphonic Acid, and Glufosinate in Human Urine Using Liquid Chromatography-Tandem Mass Spectrometry
Source: Int J Environ Res Public Health. 2022 Apr 19;19(9):4966. doi: 10.3390/ijerph19094966 (PMC9104544; doi:10.3390/ijerph19094966)
Supplement: Supplementary file 1 [file ijerph-19-04966-s001.zip › ijerph-1673361-supplementary.pdf]

Supporting information

A Method for the Analysis of Glyphosate, Aminomethylphosphonic Acid (AMPA), and Glufosinate in Human Urine Using Liquid Chromatography–Tandem Mass Spectrometry

Table S1. MRM parameters for the determination of glyphosate, AMPA, and glufosinate. For each compound SRM ion transitions are shown as *m/z* for the parent ion and a product ion. Optimized values include retention time (t_R), declustering potential (DP), entrance potential (EP), collision energy (CE), and collision cell exit potential (CXP).

**Compound**	**t_R (min)**	**Parent ion (m/z)**	**Product ion (m/z)**	**DP (V)**	**EP (V)**	**CE (V)**	**CXP (V)**	**Time (msec)**
Target compounds								
Glyphosate	4.08	168.1	63.05	-30	-10	-26	-9	300
AMPA	2.21	110.1	63.05	-50	-10	-32	-9	300
Glufosinate	3.16	180.2	62.9	-5	-10	-66	-7	300
Internal standards								
$^{13}C_2$, ^{15}N-glyphosate	4.08	171	63	-60	-10	-30	-10	300
^{13}C, ^{15}N, D_2-AMPA	2.21	114	63	-20	-10	-24	-9	300
D_3-glufosinate	3.16	183	63	-25	-10	-66	-9	300

Table S2 Improvement in signals of glyphosate, AMPA, and glufosinate following pre-cleanup using Oasis MCX cartridge prior to Oasis MAX cartridge. Pooled urine samples were fortified with 0.5 ng/mL target analytes and 10 ng/mL internal standards.

	**Peak area of Urine Samples Purified by MAX Only**	**Peak Area of Urine Samples Purified by MCX and MAX**
Glyphosate	413	4470
$^{13}C_2,^{15}N$-Glyphosate	10100	157000
AMPA	5940	8030
$^{13}C,^{15}N,D_2$-AMPA	52100	64200
Glufosinate	1200	3430
D_3-Glufosinate	28400	165000

Abbreviations: AMPA, aminomethylphosphonic acid.

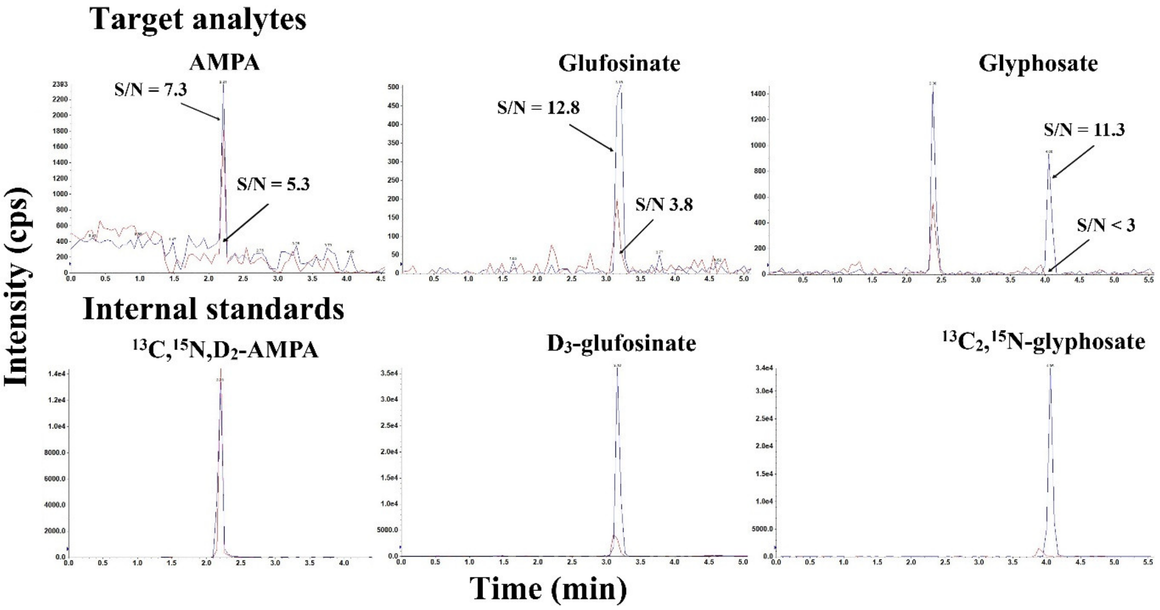

Figure S1. Overlaid chromatograms of fortified urine sample (0.5 ng/mL; internal standards: 10 ng/mL) following MAX cartridge cleanup only (red line) and the two-step procedure (MCX cartridge pre-cleanup and MAX cartridge post-cleanup) (black line). Respective signal-to-noise ratio (S/N) of the target analytes are given in the figure.
